# Ki-67 Expression by Immunohistochemistry and Quantitative Real-Time Polymerase Chain Reaction as Predictor of Clinical Response to Neoadjuvant Chemotherapy in Locally Advanced Breast Cancer

**DOI:** 10.1155/2017/6209849

**Published:** 2017-10-31

**Authors:** Prihantono Prihantono, Mochammad Hatta, Christian Binekada, Daniel Sampepajung, Haryasena Haryasena, Berti Nelwan, Andi Asadul Islam, Andi Nilawati Usman

**Affiliations:** ^1^Department of Surgery, Faculty of Medicine, Hasanuddin University, Makassar, Indonesia; ^2^Biology Molecular and Immunology Laboratory, Faculty of Medicine, Hasanuddin University, Makassar, Indonesia; ^3^Department of Pathology Anatomy, Faculty of Medicine, Hasanuddin University, Makassar, Indonesia; ^4^Halal Center, Faculty of Public Health, Hasanuddin University, Makassar, Indonesia

## Abstract

**Background:**

Chemotherapy has become a standard of treatment in managing breast cancer. To achieve proper treatment for the right patients, the predictive marker is needed. Ki-67 is a biomarker of proliferation for solid tumor. Studies mentioned association of Ki-67 expression with chemotherapy response. The study aims are to evaluate whether Ki-67 expression detected by immunohistochemistry (IHC) and quantitative real-time polymerase chain reaction (qRT-PCR) may predict clinical response to neoadjuvant chemotherapy in breast cancer.

**Methods:**

This study utilized a longitudinal study. IHC and qRT-PCR methods were used for detection of Ki-67 expression. Chemotherapy response was calculated using RECIST. Data were analyzed with Chi-square and Wilcoxon's test.

**Results:**

There were 48 subjects in this study. Analysis of Ki-67 expression with chemotherapy response has a significant correlation with *p* = 0.025 (<0.05), OR: 1.69, confidence interval (95% CI) 1.022–2.810. Analysis of Ki-67 mRNA expression with chemotherapy response has a significant correlation *p* = 0.002 (<0.05), OR: 6.85, confidence interval (95% CI) 1.064–44.193. Detection of Ki-67 expression using IHC and qRT-PCR has similar results, *p* = 0.012 (<0.05).

**Conclusion:**

These results suggest that Ki-67 expression detected by both IHC and qRT-PCR is considered to be a predictor of clinical response to neoadjuvant chemotherapy in locally advanced breast cancer.

## 1. Introduction

Breast cancer is cancer with the highest incidence in Indonesia, with an incidence of 18.6 patients in 100,000 people annually [1]. Most of the patients came in advanced stages, 63% were in stage III and stage IV by the time they were diagnosed [2]. Neoadjuvant chemotherapy has become a standard in managing locally advanced breast cancer [3].

Patients that have the same breast cancer stage and chemotherapy regiment may not have the same result. To give precise chemotherapy regimens, we need a predictive marker [4]. An ideal biomarker must differentiate tumor response towards certain chemotherapy agent before chemotherapy procedure is done so that we can avoid unnecessary therapy and toxic effect of the regiment [5]. Finding a biomarker which may predict chemotherapy response for breast cancer was still a challenge [6].

Ki-67 is a core protein which was expressed in G1, S, G2, and M phase and has been assigned as a solid tumor proliferation marker. Tumor proliferation activity shown with a Ki-67 overexpression in breast cancer is related to poor prognosis and also is predictive of neoadjuvant chemotherapy response [7]. The rate in Ki-67 relative proportion can be observed along chemotherapy and correlates with clinical and pathological response in breast cancer [8].

Breast cancer is a heterogenic disease, based on gene expression profile or breast cancer immunohistochemistry, divided into several subtypes, that is, Luminal A, Luminal B, Her-2, and triple negative. Every subtype has a different response and aggressiveness toward systemic therapy. St. Galen consensus (2011) approved that the kind of subtype influences the breast cancer chemotherapy response [9, 10].

Because of the importance of predictive marker in managing breast cancer and lack of data about Ki-67 expression in Indonesia, we are interested in evaluating the relationship of Ki-67 expression detected by IHC and qRT-PCR in breast cancer tissue prior to chemotherapy with chemotherapy response in breast cancer patients in Makassar Indonesia.

## 2. Material and Methods

### 2.1. Material

We acquired samples from breast cancer patients who received chemotherapy in Surgical Oncology Department of Wahidin Sudirohusodo Hospital, Makassar, from October 2014 until September 2015. The inclusion criteria were women with locally advanced breast cancer and invasive ductal carcinoma and women receiving cyclophosphamide-doxorubicin-5FU [11] regimen.

We performed clinicopathology data collection which involved age and grading. Then we performed immunohistochemistry panel examination of ER, PR, Her2, and Ki-67. We also detect Ki-67 expression using qRT-PCR. Chemotherapy response was measured clinically by using a caliper, at the moment before starting the 1st cycle of chemotherapy and three weeks after the 3rd cycle of chemotherapy. Data were collected, managed, analyzed, and presented in table and narration form, and then we compared them with the result of other studies.

### 2.2. Clinical Response Criteria

Clinical response in this study is classified into two categories: nonresponsive, according to RECIST, that is, stable disease or progressive disease, which is defined as reduced tumor size < 30%, the size of the tumor remaining the same, increase of the tumor size, or discovering a new tumor; responsive, according to RECIST, that is, complete response or partial response which is defined as disappearance of tumor mass or at least the reduction of tumor size by up to 30% and no new tumors.

### 2.3. Immunohistochemical Staining

Immunohistochemical staining technique is employing polymer-based methods. The primary antibodies and dilutions were used (DakoCytomation, Glostrup, Denmark): ER (clone 1D5, 1 : 100), PR (clone PgR636, 1 : 100), and Ki-67 (MIB-1, 1 : 200) [12, 13]. Paraffin blocks are cut with a thickness of 4–6 microns and placed in a special glass slide coated poly-L-lysine. Deparaffinize slides in Xylol solution 2x, every 15 minutes. Rehydrate slides in alcohol-rise 100%, 95%, 90%, 80%, and 70%, respectively, for 5 minutes, then drain.* Heat-induced antigen retrieval* (HIER): insert slides in a solution of citric acid 0.01 pH 6.0; heat in microwave for 10 minutes, and then chill. Wash slides with PBS. Block endogenous peroxidase by immersing slides in 0.3% H2O2 solution for 30 minutes at room temperature. Wash slides in PBS. Blocking normal horse serum was done by dripping a solution of 1% normal horse serum and letting it stand at room temperature for 30 minutes. Wash slides with PBS. Drip slides with primary antibody AE 1/3; enter them into the humid incubation chamber to be incubated in the room overnight. Wash slides with PBS 3 times, each for 5 minutes. Drip slides with secondary antibody solution, for 60 minutes at room temperature. Wash slides with PBS 3 times, each for 5 minutes. Drip slides with a solution of polymer-antibody-peroxidase complex, and incubate them at room temperature for 30 minutes. Wash slides with PBS 3 times, each for 5 minutes. Drip slides with DAB solution. Incubate them at room temperature.* Counterstain* with hematoxylin. Dehydrate in graded alcohol 70%, 80%, 90%, 95%, and 100%, respectively, for 3 minutes. Immerse the slides in Xylol 2x, each for 10 minutes.* Mount* and cover them with a glass deck. The results are obtained after the sample is checked by a light microscope up to 10 × 40 magnification [13, 14].

### 2.4. Immunohistochemistry Interpretation

Staining intensity and percentage of positive nuclei are recorded after manually segmenting tumor from the stroma. Tumors with ER/PR Remmele scores greater than 3 or positive nuclei greater than 1% were considered hormone receptor-positive [13]. When membrane staining is observed in >10% of tumor cells, Her2 is positive, and if less than 10% membrane staining is observed then Her2 is negative. Ki-67 is negative if there is less than 14% of nuclei staining and positive if ≥14% [13, 15].

### 2.5. Nucleic Acid Isolation

Extraction of nucleic acid from breast cancer tissue was performed using diatom guanidinium isothiocyanate (GuSCN) method. The sample volume of about 100 ug/ul breast cancer tissue was added to 900 mL of “L6” solution which consists of 120 g guanidinium thiocyanate (GuSCN) (Fluka Chemie AG, Buchs, Switzerland, cat number 50 990), 100 ml of 0.1 M Tris-HCl, pH 6.4, 22 ml 0.2 M ethylenediaminetetraacetate (EDTA), pH 8.0, and 2.6 g Triton X-100 (Packard, Instruments) with a final concentration of 50 mM Tris-HCl, 5 M GuSCN, 20 mM EDTA, and 0.1% Triton X-100. Then 20 mL diatom suspension was added which consists of 50 ml of H2O and 500 mL of 32% (w/v) “Celite” (“diatoms”) (Jansen Chimica, Beerse, Belgium, 10.846.79). 20 mL suspension of this diatom can bind 10 ug DNA tissue, and it is then vortexed and centrifuged in 1.5 ml Eppendorf tube with a speed of 13,000 rpm for 15 seconds. The supernatant was discarded, and the sediment was washed with “L2” solution consisting of 120 g GuSCN in 100 ml of 0.1 M Tris-HCl, pH 6.4, by adding 1 ml of “L2” solution. Furthermore, vortexing and centrifugation at 13,000 rpm for 15 seconds were performed; then wash the sediment 2 times with an “L2” solution, followed by washing with 1 ml of 70% ethanol 2 times and 1 ml acetone. The result is then heated in a water bath at a temperature of 56°C for 10 minutes. Then 60 mL solution of “TE” was added which consists of 1 mM EDTA in 10 mM Tris-HCl, pH 8.0; then vortexing is done and centrifugation is continued at a speed of 13,000 rpm for 2 minutes. Then the solution was incubated in the oven for 10 minutes at a temperature of 56°C. Then vortexing and centrifugation for 30 seconds at a speed of 13,000 rpm were conducted, and the supernatant was taken. The supernatant from this process will result in nucleotide extraction and was stored at −800°C before PCR analysis [16, 17].

### 2.6. Expression mRNA Ki67 by Real-Time PCR

Detection of mRNA expression of Ki-67 was done according to Real-time PCR method previously described by Mitas, 2001, and Potemski, 2006. The process of oligonucleotide primers was specific for the gene, that is, housekeeping gene GAPDH (internal control). Detection of mRNA Ki-67 gene was performed using specific primers forward and reverse PCR protocols: Ki-67 forward: TCCTTTGGTGGGCACCTAAGACCTG and Ki-67 reverse: TGATGGTTGAGGTCGTTCCTTGATG. Cycle RT-PCR for Ki-67 was 94°C for 3 minutes; 94°C for 30 seconds, 38 cycles; and the next step is PCR: 51°C for 30 seconds. Also, specific primers of housekeeping genes used GAPDH forward: TGAGTGCTGTCTCCATGTTTGA and GAPDH reverse: TCTGCTCCCCACCTCTAAGTTG. QRT-PCR used qRT-PCR Green Master Mix Kit, one stage. This protocol was optimized for instrument MX4000. Protocol was adjusted by using the instrument by changing the dye dilution based on the instruction manual and following the recommended instrument factory for RT-PCR cycle program.

Passive reference dye was included in the reaction diluted 1 : 500. The solution containing the dye was kept away from light, diluted 2x by SYBR Green QRT-PCR Master Mix, and stayed on the ice. Following the initial melting master mix, the unused portion is stored at 40°C with the following note: avoid repeating freeze-liquid cycles. Reaction experiment was prepared by adding the following components. Make a mixture of reagents to the reaction using some components such as the following: the mixture of reagents to take a final volume of 25 mL (including experimental RNA), 12.5 mL of 2x SYBR Green QRT-PCR master mix plus × mL of early primary (optimized concentration) plus nuclease—free PCR—the rate of the primary end mL H2 × (optimized concentration), 0,375 mL reference dye solution from step 1 (optional), and 1.0 mL of RT/RNase enzyme mixture 50 *μ*l total blocks with a reaction volume can also be used. The reaction was mixed gently so as not to form bubbles (do not rotate), and then the mixture is distributed to a test tube experiment by adding *x* mL RNA in each of the test tube experiments. The reaction was mixed gently so as not to form bubbles (not rotated). Reactions were centrifuged briefly, and the reaction was placed in the instrument, and the PCR program is ready to run using real-time PCR machine (CFX Connect System, Bio-Rad Laboratories, real-time PCR 96-well 0,1 ml, USA). Each sample was measured in triplicate [17–19].

### 2.7. Statistical Analysis

Data was analyzed using SPSS (Statistical Package for Social Sciences). Samples were analyzed using* Chi Square and Wilcoxon's test*. This study has an ethical approval from Health Research Ethics Committee of Medical Faculty of Hasanuddin University, RSPTN UH, dan RSUP Dr. Wahidin Sudirohusodo after lengthy discussion and evaluation, with a registration number 1659/H4.8.4.5.31/PP36-KOMETIK/2015.

## 3. Results

### 3.1. Characteristics of Respondents

The research was done in the period from October 2014 to September 2015; subjects were breast cancer patients who met the inclusion-exclusion criteria. Of all samples collected, 48 samples were examined by immunohistochemistry method and 30 samples were tested by qRT-PCR examination. Characteristics of patients were all presented with clinical stage III A and stage III B at the time of initial diagnosis: 30 patients (62.5%) who were responsive to neoadjuvant chemotherapy and 18 patients (37.5%) who were nonresponsive. Breast cancer patient characteristics are shown in Table 1.

### 3.2. A Representative of Microphotographs of ER, PR, HER2, and Ki67 Immunostaining in Invasive Ductal Carcinoma

**Figure 1:**
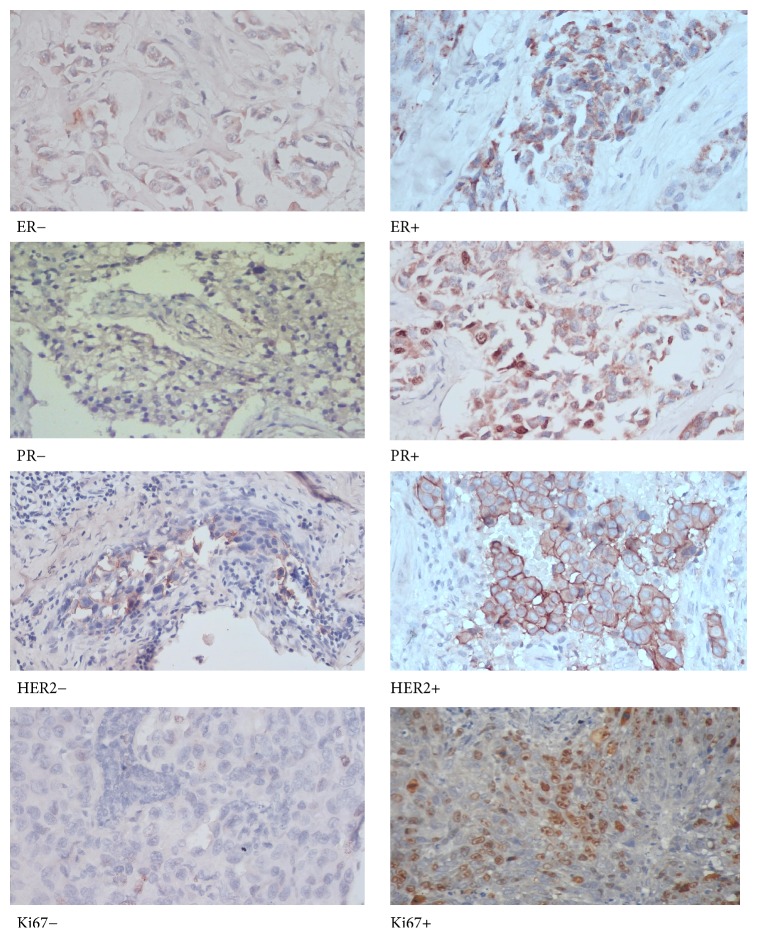
Microphotographs of ER, PR, HER2, and Ki67 immunostaining in invasive ductal carcinoma.

The representation of microphotographs of ER, PR, HER2, and Ki67 immunostaining of this research is shown in Figure 1. Microphotographs show nuclei staining in tumors with ER/PR positive, membrane staining in Her2 positive, and nuclei staining in the Ki-67 positive.

### 3.3. Amplification Curve, Melting Peak, and Melting Curve of mRNA Ki67 in Invasive Ductal Carcinoma Detected Using qRT-PCR

**Figure 2:**
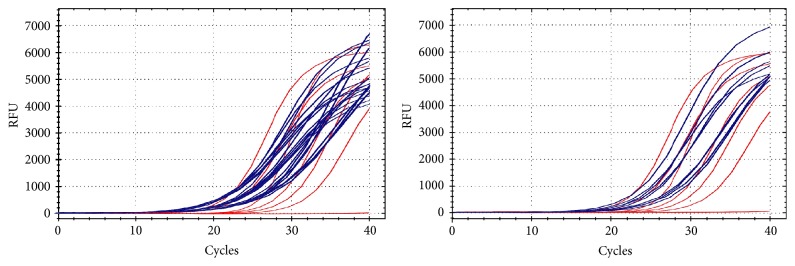
Amplification curve of Ki-67 mRNA expression.

**Figure 3:**
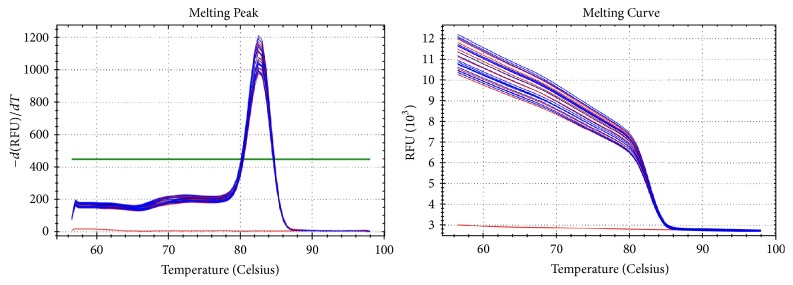
Melting peak and melting curve of Ki-67 mRNA expression.

Figures 2 and 3 show amplification curve, melting peak, and melting curve of mRNA Ki67 in invasive ductal carcinoma detected using qRT-PCR.

### 3.4. Relation of Ki-67 Expression with Neoadjuvant Chemotherapy Response

To find out whether the Ki-67 expression has a relationship with clinical response to neoadjuvant chemotherapy in breast cancer, we used bivariate analysis, which can be seen in Table 2.

Analysis of Ki-67 expression detected by immunohistochemistry found that positive Ki-67 expression tends to be responsive to neoadjuvant chemotherapy 20 (76.9%) and negative Ki-67 expression tends to be nonresponsive to neoadjuvant chemotherapy 12 (54.5%); there were statistically significant differences with *p* value = 0.025 (*p* < 0.05). This result suggests that Ki-67 expression detected by immunohistochemistry may predict clinical response to neoadjuvant chemotherapy in locally advanced breast cancer.

### 3.5. Relation of Ki-67 mRNA Expression with Neoadjuvant Chemotherapy Response

Based on qRT-PCR examination, we found that the mean value of Ki-67 mRNA expression was 11.241 ± 1.971. We determine Ki-67 mRNA expression cut-off point as 9.235, using receiver operating characteristics curve (ROC). Then Ki-67 mRNA expression was categorized as high level if it was ≥9.235 and low level < 9.235.

To find out whether Ki-67 mRNA expression has a relationship with clinical response to neoadjuvant chemotherapy in breast cancer, we used bivariate analysis, which can be seen in Table 3.

Analysis of Ki-67 mRNA expression detected by qRT-PCR found that a high level of Ki-67 mRNA expression tends to be responsive to neoadjuvant chemotherapy, 16 (76.2%), and low level of Ki-67 mRNA expression tends to be nonresponsive to neoadjuvant chemotherapy, 8 (88.9%). There were statistically significant differences with *p* value = 0.002 (*p* < 0.05). This result suggests that Ki-67 mRNA expression detected by qRT-PCR may predict clinical response to neoadjuvant chemotherapy in locally advanced breast cancer.

### 3.6. Relationship between Ki-67 Expression with Neoadjuvant Chemotherapy Response

To find out whether Ki-67 expression by immunohistochemistry has a relationship with Ki-67 mRNA expression detected by qRT-PCR, we used bivariate analysis, which can be seen in Table 4.

Analysis of Ki-67 expression by immunohistochemistry and qRT-PCR found that a high level of Ki-67 mRNA expression tends to have positive Ki-67 expression, 15 (88.2%), and low level of Ki-67 mRNA expression tends to have negative Ki-67 expression, 7 (53.8%). There were statistically significant differences with *p* value = 0.012 (*p* < 0.05). This result suggests that Ki-67 mRNA expression detected by qRT-PCR and Ki-67 expression using IHC have similar results.

### 3.7. Multivariate Analysis

Multivariate binary logistic regression analysis was used to determine independent predictors of clinical response to neoadjuvant chemotherapy in locally advanced breast cancer. Data shown in Table 5 revealed that Ki67 mRNA expression was an independent predictor for neoadjuvant chemotherapy in locally advanced breast cancer (OR, 4.385; CI, 1.206–286.53; *p* = 0.036).

## 4. Discussion

From this study, we collected variable ages: the youngest was 29 years old and the oldest was 74 years old, with a median age of 46 years old, and the most populated age found was in the 4th decade, as many as 40.5%.

Globally, breast cancer patient < 50 years old is 33% of the population; meanwhile, in Asia-Pacific, it is 42%, in South-East Asia it is 47%, and, in Australia, it is 21%. SEER data in America showed breast that cancer is common in 55–64-year-old group of age, with a median age of 61 years old [20].

Several kinds of literature mentioned that generally the age of breast cancer patient is younger in Asia than in Europe and America. This difference possibly is caused by lifestyle factors, diet pattern, or the existence of certain gene which is related to race so that the difference in age occurred [20].

From the study, data obtained are as follow low grade 16.7%, moderate grade 54.2%, and high-grade 29.2%. Histopathology grading is a particular prognostic factor. Some newest studies confirm the importance of histopathology grading as a predictive and prognostic factor in breast cancer. Engstrøm et al.'s study showed in the first five years, grade 2 and 3 breast cancer had a poorer prognosis than grade 1 [21, 22].

Breast cancer subtype is influencing chemotherapy response. Rouzier et al.'s study showed that a complete pathologic response rate in Basal-like subtype is as much as 45% and Her2 is as much as 45%; meanwhile luminal had a pathological complete response rate of 6% and no complete pathological response rate in normal-like subtype [23]. Luangdilok et al.'s study mentioned that complete pathological response in triple-negative subtype was 19.2% and Her2 was 24.2%. Meanwhile, Luminal A was 4.4%, and Luminal B was 9.7% [22]. A study of 102 breast cancer patients obtained complete pathological response in 16 (15.7%) patients. Pathological complete response that is appropriate with different subtypes is as follows: Luminal A: 0 out of 20 (0%), Luminal B: 2 out of 23 (8.7%), Her2(+): 4 out of 18 (22.2%), and triple negative: 10 out of 41 (24.4%) (*p* = 0.041) [22]. Horimoto et al.'s study mentioned that Luminal B – Her2(−) patient who received chemotherapy had a pathological complete response rate 35% which is related to disease-free survival [23, 24].

Proliferation activity has prognostic information. Measurement of proliferation activity using Ki-67 detected by IHC is still controversy [25]; whether Ki-67 scores have much prognostic information and could predict the benefit of the addition of cytotoxic chemotherapy is still a question [25–27].

Several studies had investigated Ki-67 prognostic significance in breast cancer. The study shows that Ki-67 overexpression correlates with disease-free survival and overall survival [22, 28–30]. However, a patient with high proliferation rate has a better response toward chemotherapy [8, 29]. Furthermore, this marker may help the screening of patient who might not get any advantage from chemotherapy: those who have Her2 positive and negative hormonal receptor and low proliferation tumor [8, 22, 29].

Studies revealed that Ki-67 protein expression correlates with response to chemotherapy. High Ki-67 proliferation rate was predictive of a higher probability of complete pathological response [26]. Fasching et al. investigated Ki-67 by IHC of 552 patients and found that Ki-67 expression with 13% cut-off could predict complete pathological response with 94% sensitivity and 36% specificity [13, 29]. Kim et al. found that Ki-67 expression with cutoff value 25% in breast cancer tissue is a predictor of neoadjuvant chemotherapy response. Ki-67 is also a predictive factor for complete pathological response in ER-negative and HER2-positive breast cancer patients [31]. Wang et al. found that Ki-67 independently correlated with complete pathological response and clinical response, grades, and node status. Reduction of Ki-67 expression after neoadjuvant chemotherapy was observed in patients with a relatively better response [32]. Research of Ki-67 expression measured using quantitative immunofluorescence automated quantitative analysis (AQUA) technology found that high Ki-67 levels are a predictor of neoadjuvant chemotherapy response [33].

In our previous study, we found an insignificant correlation between expression of mRNA Ki-67 baseline with chemotherapy response. But, chemotherapy cause decrease in mRNA expression of Ki-67. The rate of Ki-67 mRNA expression has a significant correlation with clinical response to chemotherapy [17, 34].

Several studies have found that changes before and after therapy in Ki-67 are a strong and independent predictor of disease-free time and survival rate [13, 35, 36]. The expression before and after chemotherapy can be a significant independent predictor of the overall survival in multivariate analysis. For this reason, nowadays, tumor response on neoadjuvant chemotherapy tryout is evaluated with the examination of immunohistochemistry Ki-67 [37].

Tumors with Ki-67 mRNA expression were examined by qRT-PCR associated with disease-free survival and overall survival of patients treated with adjuvant chemotherapy regimens. The results showed that the tumor with a high level of KI67 mRNA expression might be valuable for adjuvant therapy using docetaxel [38].

Studies found that high mRNA expression of Ki-67 was associated with a higher rate of the complete pathological response (36.4%) compared with low levels mRNA Ki-67 (5.8%). mRNA expression of Ki-67 is a predictor of the achievement of complete pathological response and better than Ki-67 expression which was detected by immunohistochemistry [39]. Ki-67 mRNA expression level is more objective and highly reproducible quantification of proliferation activity and more meaningful than Ki-67 protein expression by immunohistochemistry, either by visual scoring or by quantitative image analysis [17, 39].

## 5. Conclusion

These results suggest that Ki-67 expression detected by both IHC and qRT-PCR is considered to be predictor of clinical response to neoadjuvant chemotherapy in locally advanced breast cancer. This result also suggests that Ki-67 mRNA expression detected by qRT-PCR and Ki-67 expression using IHC have similar results.

## Figures and Tables

**Table 1:** Patient characteristics.

Characteristics	Number (%)	*p* value
Age		
≤50	27 (56.3)	0.076^*∗*^
>50	21 (43.8)	
Grade		
Low grade	8 (16.7)	0.057^*∗*^
Moderate	26 (54.2)	
High grade	14 (29.2)	
Subtype		
Luminal A	10 (20.8)	0.131^*∗*^
Luminal B	20 (41.7)	
Her2	14 (29.2)	
Triple negative	4 (8.3)	
Ki-67 (SD)		
Positive	26 (54.2)	0.025^*∗*^
Negative	22 (45.8)	
Mean of tumor size (SD)		
Before chemotherapy	12.60 (0.40)	0.031^*∗∗*^
After chemotherapy	08.06 (0.20)	

^*∗*^Chi Square statistical test; ^*∗∗*^Wilcoxon's statistical test; SD = standard deviation.

**Table 2:** Relation of Ki-67 expression with chemotherapy response in breast cancer.

Ki-67 expression	Chemotherapy response		Total
	Responsive	Nonresponsive	
Positive	20 (76.9%)	6 (23.1%)	26 (100%)
Negative	10 (45.5%)	12 (54.5%)	22 (100%)

Total	30 (62.5%)	18 (37.5%)	48 (100%)

*Chi-square*  *χ*^2^ = 5.035; df = 1; *p* = 0.025 (*p* > 0,05).

**Table 3:** Relationships between Ki-67 mRNA expression with chemotherapy response in breast cancer.

Ki-67 mRNA expression	Chemotherapy response		Total
	Responsive	Nonresponsive	
High	16 (76.2%)	5 (23.8%)	21 (100%)
Low	1 (11.1%)	8 (88.9%)	9 (100%)

Total	17 (56.7%)	13 (43.3%)	30 (100%)

*Chi-square*  *χ*^2^ = 10.866; df = 1; *p* = 0.002 (*p* < 0.05).

**Table 4:** Relationship between Ki-67 expression by immunohistochemistry and Ki-67 mRNA expression detected by qRT-PCR.

Ki-67 expression	Ki-67 mRNA expression		Total
	High	Low	
Positive	15 (88.2%)	2 (11.8%)	17 (100%)
Negative	6 (46.2%)	7 (53.8%)	13 (100%)

Total	21 (70%)	9 (30%)	48 (100%)

Spearman's *p* = 0.012 (*p* > 0,05).

**Table 5:** Multivariate regression analysis for predictive factors.

Variable	OR	95% CI	*p* ^*∗*^
Age	2.553	0.018–1.508	0.110
Grading	0.063	0.158–4.160	0.802
Subtype	0.528	0.134–2.516	0.468
Ki-67 (IHC)	0.133	0.124–20.969	0.716
Ki-67 mRNA Expression	4.385	1.206–286.53	0.036

^*∗*^Binary logistic regression analysis.
